# Evolution of policy for the treatment of substance use disorders in Qatar

**DOI:** 10.1186/s13011-021-00428-0

**Published:** 2022-01-08

**Authors:** Majid Alabdulla, Nimesh Samarasinghe, Iain Tulley, Shuja Reagu

**Affiliations:** 1 Mental Health Services, Hamad Medical Corporation, PO Box: 3050, Doha, Qatar; 2 College of Medicine, Qatar University, Doha, Qatar; 3 Weill Cornell Medicine, Doha, Qatar

**Keywords:** Qatar, Middle East North Africa, Substance abuse policy, Mental health

## Abstract

There is a marked paucity of published evidence on the extent and nature of substance use disorders in the State of Qatar. This is mirrored by a dearth of information on the policy for the treatment of substance use disorders in the public domain. Between 2007 and 2017, substance use disorders have risen from the third to leading cause of disability in Qatar. More recently, Qatar has shifted from applying a punitive only paradigm in managing substance use problems to recognizing the role of treatment and care for people with substance use disorders. Recently published official documents in Qatar define addiction as a disease and as a chronic condition where people with substance use disorders should be treated as patients who need care and assistance. This shifts the onus of providing, and developing services, for individuals with substance use disorders with healthcare providers rather than purely with the criminal justice system. Following cabinet approval, the recently established Permanent Committee for Addiction Treatment headed by the Minister of Public Health, signals the need to institutionalize systems and structures to upscale demand reduction programmes in the country. This article is a descriptive examination of the shifts in substance abuse treatment policy in Qatar, the major factors influencing this evolution, and will utilise some of the policy science theories to describe and analyse policy outcomes. The article will also frame the substance use problem in Qatar for the first time, based on documents published by various government organisations.

## Introduction

The State of Qatar, which is situated in the west coast of the Persian Gulf is home to 2.7 million inhabitants [1]. It experienced rapid economic and industrial development following the discovery of large oil fields in the 1960s and after gaining independence from the British colonial administration in 1971 [2, 3]. Qatar has also been ranked as one of the wealthiest nations, with the highest per capita GDP in the world [4]. Qatar experienced dramatic growth in population, attracting, mostly male, economic migrants from all over the world to support its massive infrastructure development leading to a resident population that is diverse racially and economically [1]. Industrialisation, rapid economic and population growth have all been associated with illicit substance use [5]. Qatar’s contemporary substance use problem and policy should therefore be examined within the context of rapid development, population growth and industrialization. These have all taken place within a short period and as such the policy shifts have been significant.

For the early part of its existence as a modern state, Qatar was able to uphold the prohibition ideology and maintain stringent law enforcement approaches to manage problems related to substance use. During this period, little emphasis was paid towards the development of drug demand reduction policies and programmes as the problem of drugs was perceived as insignificant and reasonably well contained [6]. This approach was in line with the other countries of the Middle East and North African (MENA) region where, although illicit substance use was present, its recognition as a public health issue was hampered by ideological differences and the sole responsibility of management given to the criminal justice system [7].

It is not unfair to assume, based on evidence from similar contexts, that the dramatic economic and socio-demographic changes in the country following discovery of fossil fuels influenced the pattern of substance use and the demographics of those using substances [5]. Punitive policy and stigma meant little help available locally for individuals using substances, often prompting families to seek help outside Qatar. Additionally, the authorities were faced with adverse health and behavioural problems from locals who used substances such as glue (inhalants), cannabis, alcohol, heroin and shabu (methamphetamines) and ultimately prompting a shift towards treatment for substance use disorders reaching the policy agenda and becoming a priority [8]. In parallel to this, there has been an increasing recognition of using treatment models to manage substance use disorders rather than punitive models internationally and locally [9]. Recently, there has been a shift to recognise drug use as a major public health issue and ‘Qatar has moved away from treating drug addiction merely as a criminal matter and is recognizing it as a health and social challenge and a human rights issue’ [8].

We provide an informed narrative describing the rationale for the development of Qatar’s substance abuse treatment policies, their course and outcome and the roles of the various actors and institutions already established, or which came into existence to respond to substance use disorders. Policy initiatives, including the national structures created to respond to growing concerns related to substance use are depicted chronologically in Table 1. Significant events that had an impact on policy are also included. The range of policies, initiatives and events provides a flavour of how drug policies were embedded in government activities.

**Table 1:** Policy initiatives, structures and events

1977	Bureau of Interpol and Drug (BID) established under the Criminal Investigation Department.
1980s	BID became known as the Drug and Alcohol Combating Unit.
1987	Qatar enacted first law on Control and Regulation of Narcotic Drugs and Dangerous Psychotropic Substances.
1991	Drug Enforcement Administration (DEA) established under the Ministry of Interior (MOI). DEA commence drug awareness and prevention activities.
1999	Permanent Committee for Drugs and Alcohol Affairs (PCDAA) established under the MOI.
Early 2000	Young locals using inhalants, heroin and stimulant use reached the attention of policy makers.
2006	Social Rehabilitation Centre was established by Law No 21 of 2006.
2009	Social Rehabilitation Centre commenced supporting people with drug problems and works collaboratively with the MOI.
2012	Supreme Council of Health’s Treatment and Rehabilitation Centre (TRC) established with in-patient and out-patient facilities for people who use drugs. The TRC starts receiving referrals from concerned relatives of people who use drugs and the MOI.
2013	Qatar’s Third Human Development Report states that the country’s drug problem is relatively contained.
2015	Qatar’s Fourth Human Development Reports states that addiction is a health and social challenge and a human rights issue.
2015	The Emiri Decision No (17) of 2015 paved the way to establish the Naufar Center as a state-of-the-art resort type facility to treat patients with substance use disorders. It includes inpatient, outpatient and residential rehabilitation services.
2015	The Emiri Decision No (17) of 2015 defines addiction as a disease and places the ownership of treatment for substance use disorders within Naufar Center.
2017	Qatar Institute of Health Matrix and Evaluation states that drug use disorders have causes significant disability within a ten-year period.
2018	The Qatar Second National Development Strategy includes drug control as an outcome.
2019	Cabinet approved the decision to establish a Permanent Committee for Addiction Treatment headed by the Minister of Public Health.

## External influences

The State of Qatar geographically lies within the Middle East and North Africa (MENA) region, which is known to have grown and produced many psychoactive substances. Although there are no reports of opium or cannabis being produced in Qatar, other countries within the MENA region such as Iran cultivated Opium [10]. Morocco is known to have grown cannabis [11]. Lebanon has grown small quantities of opium and produced amphetamine type substance in the form of Captagon [12]. Egypt has produced amphetamine type substances and Yemen and Somalia have cultivated khat [12]. MENA is also an area of drug consumption and transit, the latter related to MENA’s proximity to the golden crescent where opium is illicitly produced in large quantities and being at the geographical heart of drug trafficking routes [13]. Qatar’s geographical position places it as a transit country for the movement of heroin from countries in the Golden Crescent into Europe [14] some of which is leaked into the local illicit drug market [15].

Historically, substances such as opium and cannabis have been used in the Arabic and Islamic cultures during the ancient Egypt and medieval period [16, 17]. In Islamic countries, the use of mind and mood-altering substances are forbidden (*haram)* by the Holy Quran and is not culturally acceptable [17]. Nonetheless, illicit drug use is widespread in many Islamic countries throughout the world [7].

Drug control attempts go as far back as 1879 when Egypt enacted legislation to prohibit the use of cannabis [18]. Since, most countries in the MENA region are known to have adopted drug policies predominantly focusing on prohibition and stringent law enforcement, rather than adopting a balanced approach between supply and demand reduction [19]. Little emphasis has been paid towards treatment and public health, although recently, some countries have responded to the increasing demand for treatment and rehabilitation with some level of healthcare service provision [20].

There had been attempts to harmonize drug control policies in the region where 22 Arab nations that belong to the League of Arab States (LAS), including Qatar, introduced and ratified the regional Arab Convention Against Illicit Traffic in Narcotic Drugs and Psychotropic Substances in 1995 [21]. This convention echoed what the United Nations Commission on Narcotic Drugs attempted to achieve when the 1961, 1971 and 1988 UN drug control conventions were introduced. The Arab convention, in addition to supply reduction measures, advocates on drug education, treatment, rehabilitation and aftercare. The Convention also states that “the parties may provide, either as an alternative to conviction or punishment, or in addition to conviction or punishment of an offence established in accordance with paragraph 2 of this article, measures for the treatment, education, aftercare, rehabilitation or social reintegration of the offender” [21] (Arab Convention against illicit Traffic in Narcotic and Psychotropic Substances.

These regional influences gave rise to a group of legal and law enforcement experts to work collaboratively and boost drug law enforcement by sharing intelligence, and the collating, analysing and disseminating of information on drug related offences in the region. Initially, the examination of substance use problems in Qatar was solely fixated on law enforcement concerns and did not contain involvement from other groups such as health and social services. Any discussion on substance use took place in that circumscribed context. Furthermore, it took place in the backdrop of little efforts in regard to the harmonization of drug demand reduction policies and programmes in the LAS, influenced by the absence of any binding international instrument to guarantee the nature and type of substance use treatment service provision.

Later, with the increasing uncertainties and complexities associated with the management of illicit substance use among locals, gave rise to a local epistemic community [22] a group of professionals with recognized expertise and competence in the area of drug treatment and rehabilitation. The members of the epistemic community belonged to medical and non-medical professions, hired from overseas to develop local drug treatment and rehabilitation services. Their principle belief was that human welfare will improve as a result of implementation of evidence-based treatment for people with substance use disorders. They also had a causal belief in that substnace use disorders, if left untreated, can become a major public health problem. These epistemic actors’ common set of practices involved the diffusion of the medication assisted and psychosocial treatment modalitiesand included substitute prescribing which was a radical departure from total abstinence as an outcome demanded by the criminal justice system led management.

The experts within the so called Epistemic community worked with a locally appointed rapporteur in laying the foundation for the development of policies and services. This appointed rapporteur operated behind the scenes, seeking support and legitimacy from the state’s influential decision-makers, to establish a state-of-the-art drug treatment and rehabilitation center for Qatar.

## The connexion of criminal justice services

Qatar is a signatory to the United Nations Single Convention on Narcotic drugs 1961 as amended in 1972, the Convention on Psychotropic Substances of 1971 and the Convention Against Illicit Trafficking Narcotic Drugs and Psychotropic Substances 1988. These international instruments prompted the enactment of law No 9 of 1987 on Control and Regulation of Narcotic Drugs and Dangerous Psychotropic Substances in Qatar. Since, the dominant response to managing drug problems were located within the criminal justice system, which provided a stronger role for legal and law enforcement agencies in defining the drug problem as a criminal issue.

The involvement of the criminal justice system to manage drug problems date back to 1977 when a specialised office named as the Bureau of Interpol and Drug was established under the Criminal Investigation Department. In the early 1980s it was known as the Drug and Alcohol Combating Unit. Later in 1991, by a Ministerial decree, the Drug Enforcement Administration (DEA) was established under the purview of the Ministry of Interior [15]. Institutionalising international norms on drug control, particularly supply reduction policies and programs had been a key priority although the Drug Enforcement Department at the MOI also had a role in nationwide drug awareness and prevention campaigns [15]. The latter, which forms part of the demand reduction component of the Single Convention on Narcotic drugs 1961, was activated when the Drug Enforcement Administration of the Ministry of Interior launched drug prevention programs in schools and in the community at large. The DEA also provided a stronger role for the Police, which is under the purview of the Ministry of Interior, to arrest and charge people with drug related offences.

The law No 9 of 1987 on Control and Regulation of Narcotic Drugs and Dangerous Psychotropic Substances make provision for a person who use drugs to be admitted to a treatment center as an alternative to criminal sanctions. The period of mandated treatment is between 3 months to 1 year [23]. These legal provisions enable criminal justice services to mandate or coerce people to undergo rehabilitation at the Naufar Center, Qatar’s specialist substance use disorder treatment provider. Often, criminal justice system’s goal for the person mandated to treatment was abstinence and rehabilitation was seen as a means to safeguard and promote the interests and wellbeing of family members and the local community at large. Majority of policy makers representing criminal justice services viewed this as the method to bring some individuals who would not voluntarily seek treatment. It is a belief shared by some influential policy makers representing the health sector. These notions have further strengthened the role of criminal justice services in defining and managing the drug problem.

The Ministry of Interior data suggest a significant increase in the number of people who use substances from 594 in 2011 to 4202 in 2016, a nine-fold increase [8, 15]. Similarly, the number of drug offences increased from 461 in 2011 to 4175 in 2016 [8, 15]. Up until recently, the country’s drug problem had been described utilising data collected by the Ministry of Interior.

## Growth of drug treatment and rehabilitation services

The Social Rehabilitation Center established by Law No 21 of 2006, which works closely with the Ministry of Interior, commenced receiving referrals to help and support people with substance use problems in 2009. Similarly, the Supreme Council of Health’s (currently known as the Ministry of Public Health) Treatment and Rehabilitation Center worked closely with the Ministry of Interior and provided in-patient and out-patient services for people with substance use disorders. At the time, the need for treatment and rehabilitation for people who use drugs was articulated by the Ministry of Interior’s Drug Enforcement Department who established a unit to receive telephone calls from families and parents of people who use drugs [8, 15]. Drug use disorders have been described by these services as behavioural disorders where some programs introduced as ‘second chance program for addicts’ [6].

With growing demand for drug treatment and rehabilitation services, the Emiri Decision No (17) of 2015 paved the way to establish the Naufar Center, a medically led state of the art purpose built five star facility, which includes 127 residential rooms providing, in-patient, out-patient and residential services for people with substance use disorders. The Supreme Council of Health’s Treatment and Rehabilitation Center was disbanded as Naufar Center was expected to take on the services they previously provided. One of the objectives of the Naufar Center as articulated by the same Emiri decision is ‘to work on improving the image of the addict as being a patient who needs assistance and care’ [24]. This is the first time a government document defining addiction as a disease and placing the ownership of treatment for drug use disorders within the health sector. The Emir’s involvement on matters concerning drug treatment policies signifies the importance placed in the treatment and rehabilitation of people who use drugs in Qatar.

The proximity of the locally appointed rapporteur who advocated on drug treatment and rehabilitation with key stakeholders determined their influence over the establishment of Naufar Center, governed by a unique Emiri decision. Subsequently, the model of care at Naufar Center was developed and implemented with support from a group of knowledge experts sourced internationally. This marked the first clear departure from a criminal justice abstinence focused outcome to a more rehabilitation oriented health care based outcomes.

The development of services for people with substance use disorders have taken place in the absence of studies or estimates conducted on the treatment gap and harms caused by drug use in Qatar. This includes the lack of prevalence estimates on drug related HIV, Hepatitis C, overdose and mortality rates. However, the introduction of not aiming for abstinence and substitute prescribing is not without criticism, particularly from stakeholders representing criminal justice services Their belief is that only psychosocial treatment should be available for people who use drugs and that prescribing of opioid substitute medication would send a contradictory message against the prevailing prohibitionist policy and goal of a drug free society.

There also remain questions regarding the affordability and accessibility of services at Naufar Center. For example, while services are free for locals, it has been reported by internal audits that majority of non-locals are unable afford and pay for the services provided. Furthermore, the need for strong collaborative working practices between Naufar Center and the wider health system, in particular to accommodate young people and those with co-occurring disorders has been highlighted from time to time by secondary care mental health and criminal justice services.

Developing more comprehensive and accessible services have remained difficult in face of lack of epidemiological data on substance use. More so, when the population is so varied and derived from diverse nationalities. A recently published study explored Emergency Department presentations of alcohol and substance abuse across Qatar [25]. It reported alcohol as the primary substance of abuse. Interestingly, while alcohol was almost always the only substance of abuse among non-local Qataris, native Qataris had the highest proportion of complicated substance use with substances other than alcohol [25]. It will be data as such that will inform development of services with better access and coverage than what is currently available. This in addition to availability of services within primary health care has been set as the task for the newly formed Permanent Committee for Addiction Treatment purposely headed by the Minster of Public Health [26].

## Development within the national framework

The Qatar National Vision 2030, published in 2008 by the General Secretariat for Development Planning, articulates the vision at transforming Qatar into an advanced country by 2030. This document, which is considered as a roadmap to develop Qatar, identifies four pillars of development: human, social, economic and environmental. The human development section of the document, although not directly addressing the substance use problem, discusses the need to establish a comprehensive and integrated world-class healthcare system, and aspires to promote the physical and mental health of the population [6].

It has been reported that the United Nations Development Programme supported the development of Qatar’s first Human Development Report [27]. Typically, countries submit their human development reports regularly to the United Nations Development Program. These reports intend to place people at the centre of the development process, aims to address issues of national concern, suggest ideas and debate policies. Although Qatar’s Third Human Development Report states that the country’s drug problem is rather contained [28] (Ministry of Development Planning and Statistics, 2012:93), the Fourth Report challenges the status quo and makes remarkable references to the country’s drug problem and how it should be tackled [28]. The report states that;

*“Qatar has moved away from treating drug addiction merely as a criminal matter and is recognising it as a health and social challenge and a human rights issue. The right to health includes the right to obtain health services without fear of punishment. By incorporating a human rights perspective into the process of legislative reform in the laws and policies governing drugs, Qatar could improve access to treatment and reduce the prevalence of substance abusers” (Ministry of Development Planning and Statistics* [8, 28],

Furthermore, the same report contextualizes substance use among the youth as a development issue, as young Qataris are expected to play a critical role in realizing the Qatar National Vision. Young people using substances has been recognised as an obstacle to socio-economic development. Some of the ideologies articulated by the Ministry of Development Planning and Statistics have parallels with drug use disorders being considered as a major public health problem, the causal belief shared by epistemic actors who supported Naufar Center and those who advocated for drug treatment.

Framing the substance use problem as a public health and development issue in Qatar coincides with the introduction of the United Nations’ Sustainable Development Goals (SDGs) in 2015. Consequently, targets have been set to strengthen the prevention and treatment of substance use disorders in member states. Qatar is a signatory to the SDGs and contemporary official document analysis suggest that development policies and drug demand reduction efforts should work side by side to meet common goals. The need to shift the balance from punitive to care and treatment of people who use drugs had been articulated in the documents analysed.

Drug-control has been included as an outcome in the Qatar Second National Development Strategy published in 2018. The report states that drug control “was not considered a stand-alone outcome when the status quo was analysed in 2008; rather, it was implicitly incorporated under the fight against crime. However, the current threat of drugs to young people and the increased number of people who use drugsand of attempts to smuggle drugs into Qatar have necessitated the inclusion of drug control as a stand-alone intermediate outcome in order to enhance social security and safeguard national human development” [8]. Earlier in 2017, the Qatar Institute of Health Matrix and Evaluation published information denoting that drug use disorders have risen from the third to leading cause of disability within a ten-year period. Drug use disorders are also described as the number one cause of death and disability combined [29]. These findings have ensured that substance use disorder treatment and rehabilitation continue to be on the policy agenda for decision-makers to act upon.

## Conclusion

The evolution of Qatar’s policy approach to manage substance abuse disorders has now led to a degree of stability, both in its theoretical understanding of the problem and in its practice. Significantly, it has seen a shift from applying a punitive only paradigm in managing drug problems to include the provision of drug treatment and rehabilitation. The state has now placed healthcare in the driver’s seat to lead the development of a comprehensive, more accessible service with pragmatic outcomes based on international best practice.

However, without actual local data, it will not be possible to translate this policy success into positive outcomes in users of substances in Qatar. The data is emerging but needs to be part of the actual policy to highlight its priority.

Additionally, there is overwhelming evidence pointing at higher prevalence of chronic physical and mental health conditions in individuals with substance use disorders attending primary, secondary or tertiary healthcare facilities [30–32]. Lack of integration of healthcare systems and treatment programs for people with substance use disorders, leads to patients falling through gaps and missing out on cost effective care.

There is a need to establish a decentralised network of affordable and accessible drug treatment services in Qatar. Expansion of service from a single specialist service provider and the development of integrated care pathways for patients with substance use disorders within the general health system is likely be a deliberation for the Permanent Committee for Addiction Treatment. A central tenet of this development would be empowering primary and community health care in screening and brief interventions, and in the setting up of a decentralised system of providers who work closely with people who use drugs, focusing on prevention, education and treatment. An effective national system for the treatment of substance use disorders requires a coordinated and integrated response. The aim should be to deliver services and interventions in multiple settings and targeting different groups at different stages in terms of the severity of their substance use disorder. Providing effective treatment and care services for substance use disorders as part of an integrated and well-coordinated treatment system is an investment in the health of people with substance use disorders. It is also an investment in the healthy and safe development of families, communities and Qatar.

Limited local evidence on the effectiveness of services currently being delivered to substance users in Qatar has created a vacuum where some stakeholders have challenged existing treatment models and interventions, including their suitability and cultural appropriateness. In addition to basic epidemiological research, some more directed research highlighted by local needs also needs to be prioritised to raise awareness and tackle stigma.

## Data Availability

Additional and supporting data are available from the authors upon reasonable request.

## Abbreviations

GDP: Gross domestic product
MENA: Middle Eastern and North African Region
MOI: Ministry of Interior
SDG: Sustainable developmental goals
PCDAA: Permanent committee for drug and alcohol affairs
TRC: Treatment and Rehabilitation Centre
